# The Role of Emotional Entitlement, Gratitude, and Social Support in Psychological Adjustment During Radiotherapy: A Longitudinal Study

**DOI:** 10.1002/smi.70207

**Published:** 2026-07-13

**Authors:** Roni Laslo‐Roth, Sivan George‐Levi, Lior Elimelech, Yael Galin Loncich, Nirit Wiskop‐Farkash, Myriam Sultan, Eli Sapir

**Affiliations:** ^1^ School of Behavioral Sciences Peres Academic Center Rehovot Israel; ^2^ Department of Psychology Achva Academic College Yinon Israel; ^3^ Radiation Oncology Department Samson Assuta Ashdod University Hospital Ashdod Israel

**Keywords:** anxiety, depression, emotional entitlement, gratitude, life satisfaction, radiation therapy, social support

## Abstract

A cancer diagnosis is frequently accompanied by significant psychological distress, particularly during radiotherapy (RT). Given that many established risk factors for distress are largely immutable, identifying resilience resources that promote adjustment during RT is a central clinical priority. The present longitudinal study examined whether emotional entitlement, gratitude, and perceived social support predict end‐of‐treatment anxiety, depression, and life satisfaction beyond baseline psychological status. Participants were 146 cancer patients undergoing RT (Mean age = 56.17 years, SD = 11.65; 89.9% female). Data were collected at three time points across the RT regimen: at the pre‐treatment RT simulation session, during the first RT session, and at treatment completion, approximately 3–6 weeks later. Measures included anxiety and depression symptoms, life satisfaction, emotional entitlement, gratitude, and perceived social support. Results indicated that anxiety decreased and life satisfaction increased from baseline to treatment completion, whereas depressive symptoms remained stable over time. Hierarchical regression analyses were performed to predict post‐treatment outcomes while controlling baseline levels of the respective variables. Across all models, baseline psychological status emerged as a robust predictor of end‐of‐treatment outcomes. However, resilience resources contributed additional explanatory power for depression and life satisfaction. Higher emotional entitlement to positive emotions predicted greater life satisfaction and lower depressive symptoms, whereas reduced perceived friend support was associated with higher depression. Unexpectedly, higher gratitude was associated with lower life satisfaction at treatment completion. In contrast, anxiety was predicted exclusively by baseline anxiety. These findings suggest distinct predictive patterns across psychological outcomes during RT. Whereas end‐of‐treatment anxiety was predicted primarily by initial anxiety levels, depression and life satisfaction were also associated with modifiable emotional and interpersonal resources. The results underscore the importance of early psychological screening and targeted psychosocial interventions aimed at strengthening emotional resources and peer support during radiotherapy.

## 1 Introduction

Cancer is one of the leading causes of death worldwide; however, advances in early diagnosis and treatment have reduced mortality rates, resulting in a growing number of patients who must cope with the ongoing effects of cancer and its treatments (Siegel et al. 2025). Radiation therapy (RT) is a widely used cancer treatment that involves the application of high‐energy radiation to destroy malignant cells. Approximately half of all individuals diagnosed with cancer receive RT during the course of their disease, either with curative intent or as part of a multimodal treatment strategy that may include surgery and/or chemotherapy. In addition to its role in disease management, RT is frequently administered in advanced or incurable cancer to reduce symptom burden and improve quality of life (Chandra et al. 2021). Given that RT often represents the final stage of active oncologic care before patients return home, identifying factors that may contribute to patients' psychological adaptation at the end of treatment is critical for identifying individuals at risk and guiding targeted interventions aimed at long‐term adjustment to cancer (Liang et al. 2025).

A cancer diagnosis and its treatments, such as RT, substantially affect mental health and wellbeing, with reduced life satisfaction and heightening distress often arise at the time of diagnosis (Mungase et al. 2021). During RT, many patients experience elevated anxiety and depression due to fear of the machine and radiation, treatment side effects, and intensive care demands, impairing health‐related quality of life during and after treatment (Kim and Park 2021). Psychological distress is typically elevated at the onset of radiotherapy and tends to decline over time; however, a substantial proportion of patients continue to report clinically significant distress towards treatment completion (Li et al. 2025). In this later phase, depression and reduced life satisfaction become particularly salient as patients approach discharge (Li et al. 2025).

Although anxiety and depression are often examined together as indicators of psychological distress, they represent partially distinct forms of emotional suffering among cancer patients. Anxiety is typically characterised by heightened threat anticipation, uncertainty, fear, and physiological arousal, and in oncology settings it may be closely linked to treatment‐related concerns such as fear of procedures, side effects, disease progression, or recurrence (Curran et al. 2017). Depression, by contrast, is more strongly associated with sadness, hopelessness, loss of interest, reduced motivation, and diminished perceived meaning or pleasure (Pitman et al. 2018). Thus, while both persistent anxiety and depression may undermine well‐being, anxiety may reflect an ongoing response to perceived threat and uncertainty, whereas depression may reflect a more generalised erosion of positive affect, agency, and engagement with life (Hinz et al. 2019).

From a theoretical perspective, adaptation to cancer and radiotherapy can be understood through Lazarus and Folkman's well‐established transactional model of stress and coping, which conceptualises illness as a crisis involving objective clinical demands and individual vulnerabilities (Lazarus and Folkman 1984). Within this framework, emotional outcomes are shaped by situational characteristics (e.g., clinical and treatment‐related factors), personal characteristics (e.g., demographic factors), patients' cognitive appraisals of available internal and external emotional and social resources, and the coping strategies they use to manage treatment‐related demands. In the present study, emotional entitlement and gratitude are conceptualised as antecedent person‐level emotional resources, whereas perceived social support is conceptualised as an antecedent interpersonal resource that reflects patients' appraisal of available external support.

Younger age and female gender have been associated with higher levels of distress among cancer patients (Boakye et al. 2022; Silva et al. 2022), consistent with general population patterns (Niedzwiedz et al. 2019). However, other research has reported inconsistent associations for age and gender, and clinical factors such as cancer type and tumour location typically explain only a small proportion of variance in distress (Cook et al. 2018). Overall, mixed findings suggest that demographic and clinical factors alone do not consistently account for psychological outcomes in cancer patients.

Patients' positive appraisals of their emotional and social resources early in treatment may better predict emotional coping throughout (Lazarus and Folkman 1984), thereby offering additional insight into factors that contribute to reduced distress and higher life satisfaction. This aligns with broaden‐and‐build theory (Fredrickson 2001, 2025), which posits that positive emotions broaden individuals’ cognitive and behavioural repertoires and, over time, build enduring psychological and social resources that support resilience and adaptive coping. Therefore, the current study focused on entitlement beliefs to experience positive emotions, perceived social support and gratitude which may foster psychological adaptation among cancer patients.

Emotional entitlement is a multidimensional psychological construct reflecting an individual's belief in their right to experience and express emotions (Laslo‐Roth and George‐Levi 2024). It includes three main types: entitlement to positive emotions, the belief that one deserves to experience positive feelings such as happiness and joy. Entitlement to negative emotions, the right to legitimately feel and express negative emotions like sadness or anger. And uncompromised emotional entitlement, a maladaptive form characterised by rigid and exaggerated beliefs in one's right to emotional validation (Laslo‐Roth and George‐Levi 2024). While emotional entitlement to negative emotions and uncompromised emotional entitlement often linked to psychological distress and reduced life satisfaction, emotional entitlement to positive emotions reflects an adaptive form of entitlement related to higher life satisfaction, positive emotions and reduced anxiety and depression (Huang et al. 2025; Laslo‐Roth and George‐Levi 2024).

Gratitude is defined as thankfulness or appreciation of the positive in the world and is significant for human prosocial behaviour (McCullough et al. 2001). This construct has emerged as particularly important among cancer patients, promoting psychological well‐being and adaptive coping during treatment (Shariatmadari et al. 2025). Recent studies highlight gratitude interventions, such as gratitude journaling, improve psychological well‐being and resilience in patients with breast cancer undergoing radiotherapy, leading to reduced psychological distress and improved quality of life (Shariatmadari et al. 2025).

Perceived social support is a well‐established psychological construct broadly defined as an individual's subjective evaluation of the availability and adequacy of practical, emotional, and informational resources provided by others (Taylor 2011). It plays a critical role in enhancing coping abilities and psychological well‐being, particularly in the context of chronic illness and cancer (Uchino 2009). In cancer patients undergoing RT, higher perceived social support has been associated with reduced psychological distress, lower levels of depression and higher life satisfaction (Brajković et al. 2023; Li et al. 2025).

Social support is commonly described as consisting of three main types: support from friends and the broader social network, family support, and support from the medical team (Ruiz‐Rodríguez et al. 2022). Studies indicate that family support provides emotional comfort, material assistance, and a sense of belonging, which have been shown to significantly alleviate anxiety and depression in breast cancer patients while support from medical staff and friends was found to be associated with global health and functioning (Arora et al. 2007; Ruiz‐Rodríguez et al. 2022).

Perceived social support should be distinguished from received or enacted support (Uchino 2009; Taylor 2011). Perceived support refers to individuals' subjective appraisal that support would be available and adequate if needed, and is therefore often conceptualized as a relatively enduring interpersonal resource or antecedent appraisal within the transactional model. Received support, by contrast, refers to actual supportive behaviors or exchanges that occur in response to stress and may be better understood as part of the coping process itself. Thus, in the present study, perceived support from family, friends, and radiotherapy staff was examined as an appraised interpersonal resource, rather than as an indicator of actual support provision during treatment.

Although the resources examined in this study can be understood as antecedent psychosocial resources within the transactional model, they should not be viewed as fixed traits. Emotional entitlement reflects a belief system regarding the legitimacy of experiencing and expressing emotions, which may be shaped by repeated interpersonal and socialization experiences while remaining open to change through new relational experiences, therapeutic work, and increased self‐permission (Laslo‐Roth and George‐Levi 2024). Gratitude is commonly conceptualized as a dispositional tendency to notice and appreciate positive aspects of life, yet it can also be strengthened through intentional practices (Wong 2023). Similarly, perceived social support reflects patients' appraisal of the availability and adequacy of support from others; while this appraisal may be partly rooted in prior relational experiences, it may also fluctuate in response to illness‐related circumstances and actual supportive interactions (Uchino 2009). Thus, these variables represent relatively enduring but potentially modifiable psychosocial resources that may shape patients' appraisals and coping during radiotherapy.

### 1.1 The Current Study

This review examines factors associated with anxiety, depression, and life satisfaction among cancer patients undergoing radiotherapy. The study is grounded in Lazarus and Folkman's transactional model of stress and coping, which emphasises the role of situational characteristics, personal attributes, cognitive appraisals, and coping processes in shaping emotional outcomes. In the context of cancer, clinical factors (e.g., cancer stage, tumour type, family history of cancer) and demographic characteristics (e.g., age, gender) are sometimes linked to psychological morbidity, yet are largely non‐modifiable (Pitman et al. 2018).

The transactional model of stress and coping and Fredrickson's broaden‐and‐build theory offer compatible perspectives on psychological adjustment during radiotherapy. Lazarus and Folkman's (1984) model provides the broader framework for understanding how patients appraise illness‐related demands and available resources, and how these appraisals shape coping and emotional outcomes. Fredrickson's broaden‐and‐build theory (Fredrickson 2001, 2025) complements this framework by specifying how positive emotions may support adaptive coping: by broadening momentary thought‐action repertoires, positive emotions can facilitate more flexible appraisals, openness to support, and engagement with health‐supportive behaviours, thereby helping to build enduring psychological and interpersonal resources over time (Fredrickson and Joiner 2018). More recent accounts of upward spiral dynamics further suggest that these processes may become reciprocal, such that positive emotional experiences promote coping, social connection, and health‐supportive engagement, which in turn create further opportunities for positive emotions and resource building (Fredrickson and Joiner 2018; Van Cappellen et al. 2018).

This updated perspective is particularly relevant to radiotherapy, which requires patients to cope with repeated treatment sessions, uncertainty, and changing emotional demands over several weeks. Within this context, emotional entitlement to positive emotions, gratitude, and perceived social support may represent modifiable psychosocial resources that shape patients' appraisals and coping while also supporting small upward spirals of connection, engagement, and well‐being during treatment (Fredrickson and Joiner 2018).

While most research in this area has focused on distress‐related outcomes, positive indicators of adaptation such as life satisfaction have received far less attention (Phoosuwan and Lundberg 2023). Therefore, the current study focused not only on anxiety and depression as outcomes but also on life satisfaction. Accordingly, the study examines whether identification of psychological resources early in treatment predict patient life satisfaction, depression, and anxiety at the end of radiotherapy, above and beyond demographic and clinical factors and baseline levels of these outcomes.

Thus, the study hypotheses are as follows:

H 1 Higher levels of psychological resources at the beginning of treatment, including perceived social support, emotional entitlement to positive emotion, and gratitude will predict patient higher **life satisfaction** at the end of radiation therapy, beyond demographic factors and baseline life satisfaction. While emotional entitlement to negative emotions and uncompromised emotional entitlement will demonstrate opposite patterns.

H 2 Higher levels of psychological resources at the beginning of treatment including perceived social support, emotional entitlement, and gratitude will predict lower levels of **depression** at the end of radiation therapy, beyond demographic factors and baseline depression levels. While emotional entitlement to negative emotions and uncompromised emotional entitlement will demonstrate opposite patterns.

H 3 Higher levels of psychological resources at the beginning of treatment including perceived social support, emotional entitlement, and gratitude will predict lower levels of **anxiety** at the end of radiation therapy, beyond demographic factors and baseline anxiety levels. While emotional entitlement to negative emotions and uncompromised emotional entitlement will demonstrate opposite patterns.

## 2 Method

### 2.1 Participants and Procedure

This study was part of a larger longitudinal project examining psychological adaptation among cancer patients undergoing radiotherapy at the Radiation Oncology Department of Assuta Medical Centre. Recruitment occurred between January 2022 and September 2023. During this period, 1862 individuals received daily radiation treatments lasting approximately 3 to 6 weeks. Eligibility criteria included fluency in Hebrew language and a medical condition that enables completion of the questionnaires. Of these patients, 223 were screened and found eligible for participation. Among eligible patients, 66 declined participation (29.6%). An additional 11 patients were not included in the final sample because they did not complete enrolment and/or the initial T1 assessment after eligibility screening. Thus, the final T1 sample included 146 participants, reflecting a participation rate of 65.5% among eligible patients. The study was approved by the hospital ethics committee, and informed written consent was obtained from all participants.

Participants completed questionnaires at three time points: Time 1 (simulation day, prior to treatment initiation), Time 2 (first radiation session), and Time 3 (treatment completion, approximately three to 6 weeks later). At Time 1, participants completed the Hospital Anxiety and Depression Scale (HADS) and life satisfaction measure as well as emotional entitlement scale and gratitude scale. Perceived social support was measured at Time 2, HADS and life satisfaction were reassessed at Time 3. Fifty‐eight participants discontinued after Time 1, leaving 88 individuals with complete data across all time points (39.7% attrition).

Perceived social support was assessed at Time 2, the first radiation session, rather than at Time 1 in order to reduce participant burden by distributing the questionnaire battery across the early assessment points. This decision was made in light of the clinical context, as patients undergoing radiotherapy may experience fatigue, distress, and difficulty completing a large number of questionnaires at one time.

The final sample at T1 (*N* = 146) had a mean age of 56.17 years (SD = 11.65, range 32–84). Females comprised 89.9% of the sample, and males 10.1%. Marital status distribution was 36.1% married, 48.1% single, 9.0% divorced/separated, and 6.8% widowed. Participants had an average of 2.27 children (SD = 1.61, range 0–11). Cancer diagnoses were predominantly breast cancer (89.1%), followed by prostate cancer (2.9%), and smaller proportions of lung, ovarian, lymphoma, colon cancer, and multiple primaries. Disease stages ranged from 1 to 4, with 57.5% of participants at stage 1, 17.8% at stage 2, 10.3% at stage 3%, and 3.4% at stage 4. The mean number of radiation treatments received was 14.04 (SD = 5.32). Chemotherapy was received by 36.9% of participants. Regarding comorbidities, 53.5% of participants reported having no additional disease, while 46.5% reported having at least one comorbid condition.

### 2.2 Measures

*Hospital Anxiety and Depression Scale (HADS;* Zigmond and Snaith 1983). Used to assess participants' levels of anxiety and depression. HADS is a 14‐item self‐report questionnaire composed of two subscales: Anxiety (7 items) and Depression (HADS‐D). Each item is rated on a 4‐point Likert scale ranging from 0 to 3, yielding subscale scores from 0 to 21, with higher scores indicating greater symptom severity Cronbach's *α* = 0.82 (anxiety) and 0.76 (depression) at Time 1, and 0.88 (anxiety) and 0.80 (depression) at Time 3. Cronbach's *α* = 0.82 (anxiety) and 0.76 (depression) at Time 1, and 0.88 (anxiety) and 0.80 (depression) at Time 3.

*Satisfaction with Life Scale (SWLS*; Diener et al. 1985) Five‐item scale measuring global life satisfaction on a 7‐point Likert scale (1 = strongly disagree to 7 = strongly agree). Scores represent the mean across items. Cronbach's *α* was 0.85 at Time 1 and 0.87 at Time 3.

*Emotional Entitlement Questionnaire (EEQ;* Laslo‐Roth and George‐Levi 2024) Fifteen items divided into three subscales: emotional entitlement to positive emotions‐ EEP (Cronbach's *α* = 0.83), emotional entitlement to negative emotions ‐EEN (Cronbach's *α* = 0.85, and uncompromised emotional entitlement‐ EEU (Cronbach's *α* = 0.81), each rated on a 7‐point Likert scale (1 = strongly disagree to 7 = strongly agree) with higher scores indicating greater emotional entitlement.

*Perceived Social* *Support* was measured with the MSPSS (Zimet et al. 1988), including 12 items assessing perceived support from family, friends, and significant others (the perceived social support from significant others subscale was adapted for perceived support from radiotherapy staff). Items are rated on a 7‐point Likert scale. Cronbach's *α* were 0.84 (perceived family support), 0.84 (perceived staff support) and 0.94 (perceived friend support).

*Gratitude* Questionnaire‐6 (GQ‐6) (McCullough et al. 2002) is a six‐item scale measuring dispositional gratitude on a 7‐point Likert scale where scores represent the mean of items. Cronbach's *α* was 0.68.

## 3 Results

First, attrition analyses were conducted to compare participants who completed all assessments with those who discontinued participation, using all available demographic, clinical, and psychological data. Because some participants discontinued before completing all questionnaires, sample sizes varied across comparisons. For continuous variables, MANOVAs were conducted by variable set and assessment point, and categorical variables were compared using chi‐square tests. The overall multivariate effect for continuous demographic and clinical variables was not significant, *Pillai's* *Trace* = 0.049, *F*(3, 124) = 2.12, *p* = 0.101, *η*_*p*_^2^ = 0.049; however, completers were significantly older than non‐completers, *F*(1, 126) = 4.85, *p* = 0.030, *η*_*p*_^2^ = 0.037. Chi‐square analyses showed no significant differences in gender, chemotherapy, cancer stage, or cancer type, *p* > 0.05. However, completers were more likely to be married than non‐completers, *χ* ^*2*^(1, *N* = 133) = 6.58, *p* = 0.010. Regarding psychological study variables, the overall multivariate test was not significant, *Pillai's Trace* = 0.181, *F*(10, 84) = 1.86, *p* = 0.062, *η*_*p*_^2^ = 0.181. Follow‐up tests indicated that completers reported lower baseline gratitude than non‐completers, *F*(1, 93) = 4.94, *p* = 0.029, *η*_*p*_^2^ = 0.050, whereas no significant differences were found for baseline life satisfaction, anxiety, depression, emotional entitlement, or perceived social support variables.

A repeated measures MANOVA was conducted to examine changes in psychological outcomes from baseline (T1) to post‐treatment (T3). The multivariate effect of time was significant*, F*(3, 84) = 3.10, *p* = 0.031, *η*_*p*_^2^ = 0.10, indicating an overall change in psychological functioning over the course of radiotherapy. Univariate analyses (Table 1) revealed a significant decrease in anxiety, *F*(1, 86) = 6.37, *p* = 0.013, *η*_*p*_^2^ = 0.07, and a significant increase in life satisfaction, *F*(1, 86) = 5.27, *p* = 0.024, *η*_*p*_^2^ = 0.06. In contrast, the change in depression was not statistically significant, *F*(1, 86) = 2.56, *p* = 0.113.

**TABLE 1:** Changes in psychological outcomes from baseline (T1) to the end of Treatment (T3).

Variable	Time 1 mean (SD)	Time 3 mean (SD)	F(1, 86)	*p*	Partial η^2^
Depression	0.65 (0.53)	0.58 (0.51)	2.56	0.113	0.03
Anxiety	1.04 (0.54)	0.93 (0.58)	6.37	0.013	0.07
Life satisfaction	5.56 (0.98)	5.72 (1.00)	5.27	0.024	0.06

Descriptive statistics and Pearson correlation analysis that revealed several significant associations between study variables, as can be seen Table 2.

**TABLE 2:** Descriptive statistics and correlations among study variables.

Variable	1	2	3	4	5	6	7	8	9	10	11	12	13
1. EEP^a^	—												
2. EEN^b^	0.41**	—											
3. EEU^c^	−0.03	0.04	—										
4. Gratitude	0.43**	0.30**	−0.19	—									
5. Life satisfaction T1	0.14	0.06	−0.09	0.39**	—								
6. Anxiety T1	−0.23**	−0.03	0.09	−0.18*	−0.40**	—							
7. Depression T1	−0.15	−0.10	0.16	−0.28**	−0.36**	0.50**	—						
8. Perceived staff support	0.11	0.11	0.05	0.21*	0.13	0.20*	0.09	—					
9. Perceived family support	0.30**	0.09	0.08	0.30**	0.40**	−0.20*	−0.16	0.28**	—				
10. Perceived friend support	0.16	0.23*	0.06	0.21*	0.35**	−0.21*	−0.26**	0.13	0.52**	—			
11. Life satisfaction T3	0.24*	−0.01	−0.03	0.32**	0.79**	−0.41**	−0.41**	0.14	0.42**	0.35**	—		
12. Anxiety T3	−0.29**	0.01	0.22*	−0.14	−0.25*	0.77**	0.37**	0.06	−0.19	−0.22	−0.42**	—	
13. Depression T3	−0.35**	−0.05	0.14	−0.31**	−0.34**	0.53**	0.70**	0.00	−0.25*	−0.48**	−0.54**	0.64**	—
M	6.75	5.83	2.88	6.13	5.50	1.05	0.67	4.92	6.42	6.11	5.72	0.93	0.58
SD	0.65	1.30	1.34	0.84	0.95	0.54	0.52	1.67	0.92	1.27	1.00	0.58	0.51

^a^ EEP = emotional entitlement to positive emotions.

^b^ EEN = emotional entitlement to negative emotions.

^c^ EEU = uncompromised emotional entitlement.

* *p* < 0.05.

** *p* < 0.01.

### 3.1 Predictors of Psychological Outcomes

To identify predictors of psychological outcomes at the end of treatment (T3) (beyond their initial level at baseline), three hierarchical multiple regression analyses were conducted with life satisfaction, depression, and anxiety all at time 3 as the dependent variables. For each model, demographic and clinical characteristics (age, gender, comorbidities with another disease, chemotherapy, cancer stage, amount of radiation, and family history) along with baseline levels of the respective outcome variable (T1) were entered in Step 1. In Step 2, psychological predictors were added, including the three subscales of perceived social support (perceived support from family, friends, and radiotherapy staff), the three subscales of emotional entitlement (EEP, EEN, and EEU), and gratitude.

### 3.2 Life Satisfaction

At the first regression, life satisfaction at T3 served as the dependent variable. In step 1 the model including demographic and clinical variables and baseline life satisfaction was statistically significant, *F*(8, 45) = 15.29, *p* < 0.001, and explained 73.1% of the variance in satisfaction at T3. Baseline satisfaction was the only significant predictor (*β* = 0.854, *p* < 0.001). In Step 2, adding the psychological predictors (the three subscales of perceived support, the three subscales of emotional entitlement and gratitude) significantly improved the model, Δ*R* ^*2*^ = 0.087, *F*(7, 38) = 2.60, *p* = 0.027. The final model was statistically significant, *F*(15, 38) = 11.40, *p* < 0.001, and explained 81.8% of the variance in life satisfaction at T3. Baseline satisfaction (*β* = 0.968, *p* < 0.001), EEP (*β* = 0.272, *p* < 0.05), and gratitude (*β* = −0.358, *p* < 0.01) were significant predictors, indicating that higher baseline satisfaction, higher EEP, and lower gratitude predicted higher satisfaction at T3. The data are shown in Table 3.

**TABLE 3:** Hierarchical regression analysis for predicting life satisfaction at time 3.

Predictor	Step 1 *β*	Step 2 *β*
Age	0.010	0.015
Gender	0.066	0.071
Comorbidity	0.030	0.041
Chemotherapy	−0.015	0.010
Cancer stage	0.086	0.153
Number of radiations	0.041	0.092
Family history	−0.056	−0.122
Life satisfaction T1	0.854***	0.968***
Perceived medical staff support	—	0.088
Perceived family support	—	0.068
Perceived friend support	—	−0.128
EEP^a^ T1	—	0.272*
EEN^b^ T1	—	0.012
EEU^c^ T1	—	−0.026
Gratitude T1	—	−0.358**
*R* ^2^	0.731	0.818
Model p	0.001	0.027

^a^ EEP = emotional entitlement to positive emotions at time 1.

^b^ EEN = emotional entitlement to negative emotions at time 1.

^c^ EEU = uncompromised emotional entitlement at time 1.

^*^ *p* < 0.05.

^**^ *p* < 0.01.

^***^ *p* < 0.001.

## 4 Depression

At the second regression depression at T3 served as the dependent variable. In step 1 the model including demographic and clinical variables and depression base levels was statistically significant, *F*(8, 45) = 7.67, *p* < 0.001, and explained 57.7% of the variance in depression at T3. Baseline depression was the only significant predictor (*β* = 0.738, *p* < 0.001). In Step 2, adding the psychological predictors significantly improved the model, Δ*R* ^*2*^ = 0.154, *F*(7, 38) = 3.12, *p* = 0.011. The final model was statistically significant, *F*(15, 38) = 6.89, *p* < 0.001, and explained 73.1% of the variance in depression at T3. Baseline depression (*β* = 0.577, *p* < 0.001), EEP (*β* = −0.404, *p* < 0.01), and perceived friends' support (*β* = −0.279, *p* < 0.05) were significant predictors, such that higher baseline depression, lower EEP, and lower perceived friends' support predicted higher depression at T3. The data are shown in Table 4.

**TABLE 4:** Hierarchical regression analysis predicting depression at time 3.

Predictor	Step 1 *β*	Step 2 *β*
Age	0.034	0.024
Gender	0.052	0.102
Comorbidity	0.017	−0.062
Chemotherapy	0.072	0.022
Cancer stage	−0.076	−0.098
Number of radiations	−0.091	−0.102
Family history	−0.062	−0.010
Depression T1	0.738***	0.577***
Perceived medical staff support	—	−0.009
Perceived family support	—	0.069
Perceived friend support	—	−0.279*
EEP^a^ T1	—	−0.404**
EEN^b^ T1	—	0.116
EEU^c^ T1	—	0.004
Gratitude T1	—	0.018
*R* ^2^	0.577	0.731
Model *p*	0.001	0.011

^a^ EEP = emotional entitlement to positive emotions at time 1.

^b^ EEN = emotional entitlement to negative emotions at time 1.

^c^ EEU = uncompromised emotional entitlement at time 1.

^*^ *p* < 0.05.

^**^ *p* < 0.01.

^***^ *p* < 0.001.

## 5 Anxiety

At the third regression analysis, anxiety at T3 served as the dependent variable. In Step 1, the model including demographic and clinical variables and anxiety base levels was statistically significant, *F*(8, 45) = 12.31, *p* < 0.001, accounting for 68.6% of the variance in anxiety. Among the predictors, baseline anxiety emerged as the only significant predictor (*β* = 0.824, *p* < 0.001). In Step 2, adding the psychological predictors (the three subscales of perceived support, the three subscales of emotional entitlement and gratitude) did not significantly improve the model, Δ*R* ^*2*^ = 0.038, *F*(7, 38) = 0.74, *p* = 0.641. The final model was statistically significant, *F*(15, 38) = 6.65, *p* < 0.001, and explained 72.4% of the variance in anxiety at T3. Baseline anxiety remained the strongest predictor (*β* = 0.776, *p* < 0.001), whereas none of the psychosocial predictors were statistically significant. The data are shown in Table 5.

**TABLE 5:** Hierarchical regression analysis predicting anxiety at time 3.

Predictor	Step 1 *β*	Step 2 *β*
Age	0.056	0.101
Gender	0.113	0.096
Comorbidity	−0.079	−0.126
Chemotherapy	0.147	0.129
Cancer stage	0.059	0.098
Number of radiations	0.040	0.005
Family history	−0.046	0.037
Anxiety T1	0.824***	0.776***
Perceived medical staff support	—	−0.038
Perceived family support	—	0.096
Perceived friend support	—	−0.120
EEP^a^ T1	—	−0.131
EEN^b^ T1	—	0.040
EEU^c^ T1	—	0.151
Gratitude T1	—	0.102
*R* ^2^	0.686	0.724
Model *p*	0.001	0.641

^a^ EEP = emotional entitlement to positive emotions at time 1.

^b^ EEN = emotional entitlement to negative emotions at time 1.

^c^ EEU = uncompromised emotional entitlement at time 1.

*p < 0.05.

**p < 0.01.

^***^ *p* < 0.001.

## 6 Discussion

The primary objective of this study was to identify psychological resources that may function as resilience factors, associated with lower psychological distress and higher life satisfaction, among cancer patients during radiotherapy. Emphasising predictors that operate beyond initial baseline symptoms allowed for a clearer understanding of protective factors contributing to adaptive psychological adjustment among cancer patients over time.

Mean levels of life satisfaction slightly increased from Time 1 to Time 3, whereas anxiety levels decreased over the same period, indicating a trend of improvement in overall well‐being as treatment progressed. These patterns are broadly consistent with recent longitudinal research in oncology populations showing while anxiety often decreases as patients adapt to treatment trajectory (Wang et al. 2025), changes in depressive symptoms are often more modest among cancer patients (Lee et al. 2023), as was found in the current study. While life satisfaction is rarely measured directly during cancer treatment, longitudinal improvements in psychological well‐being and quality of life are widely documented and are theoretically consistent with increased subjective life satisfaction as patients adapt over time (Chuang et al. 2024).

At the same time, clinical and demographic variables in the present study did not emerge as significant predictors of psychological outcomes at the end of treatment beyond baseline levels. This pattern suggests that demographic and clinical characteristics primarily reflect relatively stable conditions rather than dynamic processes that shape adjustment trajectories during cancer treatment. Consistent with Lazarus and Folkman's transactional model of stress and coping (Lazarus and Folkman 1984) and empirical findings in oncology populations (Naser et al. 2021), the present findings suggest that psychological adjustment during radiotherapy cannot be understood solely in terms of objective characteristics of the individual or the stressor. Rather, end‐of‐treatment outcomes appear to be associated with antecedent psychosocial resources, including person‐level emotional resources such as emotional entitlement and gratitude, and appraised interpersonal resources such as perceived social support. These resources may shape patients' cognitive appraisals of treatment‐related demands and available resources, as well as the coping processes through which they manage these demands.

The results demonstrated that life satisfaction at the end of radiotherapy, reflecting patients' positive cognitive appraisal of their lives as fulfiling and satisfactory despite adversity, was significantly predicted by baseline life satisfaction and emotional entitlement to positive emotions. According to Fredrickson's broaden‐and‐build theory, positive emotions facilitated by beliefs in one's right to positive feelings expand cognitive resources and build enduring personal assets, enabling cancer patients to maintain a sense of life satisfaction even during challenging radiotherapy (Fredrickson 2001; Garnefski and Kraaij 2018). This finding aligns with recent literature indicating that the capacity to experience positive emotions amid cancer contributes to psychological well‐being and, by extension, to life satisfaction beyond demographic and clinical factors (e.g., Liang et al. 2025).

The present findings may also be interpreted in light of more recent formulations of upward spiral dynamics (Fredrickson and Joiner 2018; Fredrickson 2025; Van Cappellen et al. 2018). Emotional entitlement to positive emotions may reflect a form of self‐permission to notice, experience, and pursue positive emotional states even during illness‐related adversity. Such self‐permission may be especially important during radiotherapy, where opportunities for positive affect are often brief and embedded within a stressful medical routine. From the perspective of the revised upward spiral theory of lifestyle change, positive emotions experienced in the context of coping or health‐supportive behaviours may increase the likelihood that patients continue to engage in such behaviours, thereby strengthening adaptive resources over time (Fredrickson and Joiner 2018). Thus, patients who feel more entitled to positive emotions may be more likely to seek moments of relief, connection, pleasure, or meaning during treatment, which may gradually support higher life satisfaction and lower depressive symptoms.

Gratitude negatively predicted life satisfaction at the end of treatment in the regression analyses, despite its positive bivariate association. This apparent inconsistency may reflect the shared variance between gratitude and the other positive resources in the analysis. When the positive, affirming aspects of gratitude are statistically controlled, the remaining variance may capture more ambivalent components of gratitude, such as awareness of unmet needs or feelings of indebtedness. Previous research has highlighted the multidimensional nature of gratitude, suggesting that its distinct facets can have differential effects on well‐being (e.g., Zhang et al. 2022). These findings underscore the importance of disentangling overlapping psychological resources to better understand their unique and sometimes counterintuitive contributions to life satisfaction.

Depression levels at the end of treatments were predicted by baseline depression, perceived friend support, and entitlement to positive emotions. The association between emotional entitlement to positive emotions and lower depressive symptoms is consistent with the broaden‐and‐build theory of positive emotions, which posits that psychological protection arises not only from the reduction of distress but also from the capacity to experience positive emotions that build enduring psychological resources (Fredrickson 2001).

Previous studies have also suggested the potential contribution of perceived friend support to reducing depressive symptoms (Arora et al. 2007). In the context of chronic illnesses such as cancer, friends offer voluntary and authentic relationships that foster emotional well‐being, whereas perceived family support tends to be more instrumental or logistical, focused on caregiving (Ruiz‐Rodríguez et al. 2022). Moreover, family dynamics can sometimes evoke feelings of dependence or conflict due to obligatory roles, while friendships represent freely chosen connections with greater potential for emotional protection (Rook 2018). Additionally, the majority of our sample consisted of women, among whom perceived friend support has been found especially salient and protective against depression (Kawachi and Berkman 2001).

This interpretation is also consistent with recent work on positivity resonance (Prinzing et al. 2023), suggesting that shared positive emotional experiences may contribute uniquely to perceived meaning in life. In the context of radiotherapy, perceived support from friends may therefore provide more than instrumental assistance; it may create moments of shared positivity, normalcy, and connection that protect against depressive symptoms.

Anxiety at the end of treatment was predicted solely by anxiety at baseline. This may reflect anxiety's more acute and state‐dependent nature during radiotherapy, where symptoms fluctuate with treatment uncertainties. The positive correlation observed between anxiety and perceived support from the medical staff without significant predictive validity may indicate that patients experiencing higher levels of anxiety actively seek more support from healthcare providers rather than receiving emotional buffering from it. This pattern has been documented in cancer care, where anxious patients are more likely to request and utilise support services as a response to heightened distress, reflecting a reaction to ongoing threat and uncertainty rather than protective effects of the support itself (Zingler et al. 2025).

The different pattern observed for anxiety and depression is clinically meaningful. Anxiety during radiotherapy may be especially sensitive to immediate treatment‐related uncertainty and perceived threat (Curran et al. 2017); therefore, patients' initial anxiety level may strongly shape their subsequent anxiety trajectory. This may explain why baseline anxiety was the only significant predictor of end‐of‐treatment anxiety in the present study. Depression, however, may be more closely related to the availability of emotional and interpersonal resources that help patients preserve positive affect, social connection, and a sense of engagement with life. This distinction is consistent with our findings showing that depressive symptoms, but not anxiety, were additionally predicted by emotional entitlement to positive emotions and perceived support from friends.

### 6.1 Limitations

This study has several important limitations. First, all data were collected via self‐report questionnaires, which are vulnerable to biases such as social desirability and recall inaccuracies, potentially affecting validity. Second, several sample‐related limitations should be acknowledged. The sample size was relatively modest and the sample was predominantly female; therefore, although sufficient for the regression analyses, the sample characteristics may limit the statistical power to detect smaller effects and constrain the generalisability of the findings, particularly to male patients undergoing radiotherapy. In addition, the relatively high attrition rate may have introduced some selection bias, as completers were older, more likely to be married, and reported lower baseline gratitude than non‐completers. These differences should be considered when interpreting the findings, although demographic variables were statistically controlled in the main regression analyses. Finally, because patients who declined participation did not provide informed consent, their demographic and clinical information was not collected or analysed for research purposes; therefore, we could not determine whether they differed from patients who enroled in the study.

Third, outcomes were assessed specifically at treatment completion, leaving uncertainty about the long‐term durability of these psychological resources beyond the immediate post‐radiotherapy period. Fourth, the sample consisted exclusively of patients receiving radiotherapy and was predominantly composed of women with breast cancer. Future research should address larger, more heterogeneous samples and examine whether these psychological resource effects generalise across different treatment modalities and diverse patient populations.

Another limitation is that time from diagnosis was not systematically assessed. Because patients may be referred to radiotherapy at different points in the cancer trajectory and following different treatment sequences, the sample likely included substantial heterogeneity in the time elapsed since diagnosis. Future studies should include this variable to better understand how illness timing may shape psychological adjustment during radiotherapy.

### 6.2 Theoretical and Practical Implications

Clinically, these results suggest that psychosocial oncology interventions could enhance adjustment by cultivating positive psychological assets while also capitalising on early indicators of risk. Entitlement to positive emotions, reflecting individuals' perception of their worth and deservingness of positive outcomes, may be developed through cognitive behavioural approaches aimed at strengthening self‐compassion and adaptive self‐permission (Riley et al. 2022). Gratitude interventions, acknowledging their complex role in cancer contexts, should emphasise multifaceted appreciation practices (Cousin et al. 2024). The significant predictive role of perceived friend support for depression raises questions about whether beneficial support derives from formal peer groups or significant personal friendships from patients' home networks, warranting future clarification. Notably, as perceived rather than enacted support, interventions could enhance recognition and identification of supportive friends, potentially renewing meaningful social connections without risking support provision paradoxes, particularly among younger patients (Sampson et al. 2025).

In line with the revised upward spiral theory of lifestyle change (Fredrickson and Joiner 2018), psychosocial interventions during radiotherapy may be most effective when they help patients experience positive affect within everyday coping and health‐supportive behaviours, rather than treating positive emotions as abstract outcomes. For example, brief interventions may encourage patients to identify small rewarding routines around treatment days, engage in meaningful contact with friends, or practice savouring and appreciation in ways that feel authentic rather than obligatory (Tian et al. 2024).

Anxiety linked more to acute treatment stressors and medical uncertainty, may require targeted psychoeducation and symptom‐focused interventions (Curran et al. 2017). In this sample, baseline levels of life satisfaction, depression, and anxiety emerged as the strongest predictors of their respective outcomes at the end of treatment, indicating that these indices function more as stable trajectories than transient reactions to treatment. This pattern suggests that early psychosocial screening pre‐treatment can reliably identify patients who are likely to experience persistent distress or reduced life satisfaction over the course of radiotherapy, enabling clinicians to allocate preventive and more intensive support to those at greatest risk from the outset.

## 7 Conclusions

The present findings suggest distinct predictive patterns of psychological adjustment during radiotherapy. While anxiety outcomes were primarily associated with baseline distress, depressive symptoms and life satisfaction were also associated with modifiable emotional and interpersonal resources. Emotional entitlement to positive emotions and perceived support from friends emerged as relevant resilience factors. These results underscore the importance of early psychological screening and targeted psychosocial interventions aimed at strengthening emotional resources and perceived peer support during radiotherapy.

## Funding

The authors have nothing to report.

## Ethics Statement

The study was approved by the Ethical Committee of the Asota Helsinki Committee, Approval number: AAA‐0099‐22.

## Consent

Participants provided written consent to participate in the study.

## Conflicts of Interest

The authors declare no conflicts of interest.

## Data Availability

The data set is available via link: https://osf.io/yf58t/overview?view_only=8270258bdc5b4bafbd6cb04d35731d64.
